# Pennsylvania

**Published:** 1899-06

**Authors:** 


### SOCIETY PROCEEDINGS.

### PENNSYLVANIA STATE VETERINARY MEDICAL ASSOCIATION.

(Concluded from page 322.)

### President's Address and Report of Committee on Legislation.[1]

*Address of the President.*

We have reason to congratulate ourselves on the continued steady and conservative growth of our Association. Taking the last three meetings, I find the addition to our membership in one year and a half to be about thirty, a good showing for our Association, composed entirely of professional men, and which has been in existence for over fifteen years since its organization.

I would say for the benefit of those gentlemen who were unable to be present at our semi-annual meeting in Pittsburg last fall, that the meeting was a most successful one. For a semi-annual meeting the attendance was very large, the papers contributed were able and thoroughly practical, their discussion full of interest and instruction, while our reception and entertainment by the brethren of the "Smoky City" was the very height of hospitality, and could not be surpassed.

On January 12, 1899, I received a letter from Dr. Hoskins calling my attention, as President of the Association, to the importance of taking some official action in relation to the appointment of a State veterinarian. In a similar emergency in 1895 a special committee of this Association was appointed to prepare resolutions and present them to Governor Hastings. These resolutions stated the important qualifications that are necessary in an efficient State veterinarian; the importance to the State of having the work of this office in suitable hands, and the desire of this organization to assist the Governor in the selection of a suitable person. Taking this as a precedent, and immediate action being necessary, Governor Stone's inauguration taking place in a few days and the possibility of him acting on this appointment very soon thereafter, as President of this Association I appointed a special committee to wait upon the Governor for the purpose of presenting the whole subject to him in its proper relations. The members of this committee were Dr. W. Horace Hoskins, Dr. James B. Rayner, Dr. J. C. McNeil, Dr. W. H. Ridge, Dr. J. W. Sallade, and the President of the Association. A committee having been appointed by the Keystone Veterinary Association to act in conjunction with the two committees met in Harrisburg on February 1st, and, after a conference in reference to the appointment, waited on Governor Stone and respectfully urged the reappointment of Dr. Leonard Pearson to the office of State Veterinarian, he being the unanimous choice of both Associations and a gentleman in whom the profession and public had perfect confidence.

As an association of veterinarians we cannot too highly commend the work of the State Livestock Sanitary Board in its efforts to eradicate from and restrict the extension of tuberculosis among the herds of our State, It is true, good work has also been done by the board in suppressing other virulent contagious diseases, but no other contagious disease is so widespread among our domestic animals, while its insidious nature in the latent and chronic form, as well as the danger of its communicability to the human family through the consumption of the products from diseased animals, gives it an importance belonging to no other disease. A circular issued by the board says: "The total value of neat-cattle of Pennsylvania amounts to forty million dollars, and all of the farm animals of this State are worth approximately one hundred and twenty-five million dollars. The total losses resulting from diseases that are probably preventable amount, conservatively estimated, to more than six million dollars. Much of this total results from the ravages of tuberculosis." In view of this statement and in consideration of the importance of this question, as well as the magnitude of the interests involved, surely a liberal appropriation by the State for the purpose of investigating and putting in operation the best methods for eradicating this disease would be money well invested. The governments of Western Europe employ various methods for controlling tuberculosis among cattle. Of these systems the Danish appears to be the most effective. In Great Britain until recently no organized effort has been made for its supression. A death-rate of 60,000 people annually from consumption has awakened England to the necessity of taking measures to abate this scourge. On December 20th last a meeting was held in London to further the objects of the National Association for the Prevention of Consumption. This meeting was addressed by the Prince of Wales, two noblemen, and the most eminent medical men in England. While the financial object of the association is more especially to educate public opinion with regard to the contagious nature and conditions which favor the extension of tuberculosis, and thereby incite communities to take all available means to arrest its ravages, it is also designed to stamp out as far as possible tuberculosis in cows, one of the chief sources of this giant evil.

The several outbreaks of rabies in this State, and the difficulty in at once stamping out this dangerous malady, through insufficient authority on the part of the State Livestock Sanitary Bard to quarantine all animals in affected districts, shows the necessity for legislation conferring this power on the Board in order that such immediate effective action may be taken toward its suppression. As a rule, the origin of these outbreaks can usually be traced to a common centre, and a prompt quarantine of all dogs in affected localities would stop extension of the disease and prevent further mischief being done.

The veterinary profession in this country cannot but appreciate the movement, led by the American Veterinary Medical Association, to secure a higher social status to veterinarians in the United States Army, in urging all members of the profession to write to their Senators and Congressmen requesting them to vote for that section of the Army Reorganization Bill giving army veterinarians the rank of lieutenant. The anomalous position occupied by the army veterinarian, partly military and partly civilian, and without proper official rank to enforce his orders, cannot be otherwise than detrimental to this branch of the cavalry service.

I thank you for your courtesy and attention, and shall not detain you further when you have so many interesting demonstrations to witness and so many papers on the programme contributed by gentlemen of well-known ability in our profession, the discussion of which necessarily requires time, and which I have no doubt will prove interesting and instructive to all of us.

GEORGE B. JOBSON, V.S.,
Franklin, Pa.

*Report of Committee on Legislation.*

Your Committee on Legislation have not placed any legislative matter before the present session of our State Legislature looking to changes in the present law regulating veterinary practice in Pennsylvania, as our present law is gradually working out the problem of the higher education and training of those entering the profession, and but few violations of the law have come to our notice during the past six months.

Some of the legislation already proposed at Harrisburg is worthy of our interest and assistance, while there have been other bills introduced that should have our earnest opposition and condemnation.

The bill introduced by the milk-inspection department of Philadelphia, looking to more stringent regulations regarding the commercial character and value of this product is destined to give greater police power to the authorities in dealing more summarily with offenders failing to comply with the standard of requirements, but it leaves open the doors that, above all else, should be protected—the source of supply, the condition of the animals, the environments under which the supply is gathered and prepared for shipment. It may have the effect of forcing dealers to lay more stringent requirements upon the shoulders of the producers, in better feeding, more uniform rations and care of their milk-producing animals, but it will not add one additional safeguard in protecting us from the dangers of an unguarded source of production. Legislation of this character is not educational, but is wholly of a police character, and is usually obnoxious and difficult of enforcement.

Another bill which savors strongly of class legislation, and which will surely fail of passage, is that proposing to establish a rigid inspection service over every storage-house and receiving-station of Western-dressed beef. In other words, it is a blow at the Western packing-houses, without any similar safeguards for the many places in our State where the slaughtering and storage of meats is carried on within our own limits. Such legislation is doubtful in propriety, because it is a legislative method of suppressing competition, and of attempting to support a no longer profitable industry in our State when conducted on the indiscriminate plan now existing, and exempt from any adequate system of inspection that would give our home-dressed meats a preference and better price in our markets. It is wholly suggested in the interest of stifling trade, and now that a great portion of our people have learned that the signs, placards, bills, and illuminated awnings about our meat-shops, advertising city-dressed meats is a delusion and snare, and that for many years hundreds of our people have been consuming Western-dressed beef under the impression that they were eating only home-raised and dressed products. The deception practised has finally brought the result that most people are willing to eat the Western meat-products, since the price has specially commended it, and the failure to provide any adequate system of safeguards at home. There can only be safety and wisdom in providing a system that shall place all meats on the same basis, requiring a thorough inspection before killing, during slaughter, and preparatory to placing on the stalls for sale, and the presence of our official stamp that shall enable the utmost novice to select properly inspected meats and exercise a choice in the purchase of home-dressed or Western-dressed meats.

A bill has been introduced and referred to the Committee on Agriculture to repeal the interstate inspection law of all dairy cattle. This bill would greatly embarrass the work of the Livestock Sanitary Board, reopen our State to the dangers of becoming a dumping-ground for tuberculous cattle, retard the work of eliminating tuberculosis from our dairies, make nugatory the work in this direction by our State Livestock Sanitary Board, and impose a burden upon our livestock owners and breeders, add danger to the physical welfare of every man, woman and child of our Commonealth, and relieve no class of our people of any hardships or burdens that should not be cheerfully borne in the interests of public health. Its only benefits would be to some dealers in cattle, who may find comfort in the thought that additional profits would be added by selling tuberculous animals to their unsuspecting clients, and add something to the demand for new stock to replace the yearly loss from those that die or become worthless and are sold for bologna from this white scourge of the earth; every good citizen, every scientifically informed member of the profession should strongly urge the defeat of this measure and bring to the public's notice the willingness of some of our people to gain commercial advantage over the victims of this disease that annually carries off fourteen out of every hundred deaths in the civilized world, not to attempt to estimate the endless suffering and misery caused by tuberculosis in all its many allied forms.

Another bill has been presented destined to relieve from any responsibility, pecuniary or otherwise, any person destroying any unmuzzled dogs in districts of our State under quarantine for rabies. The bill, should it pass, is destined to meet with opposition from many people, and likely to run the gauntlet of the courts as to its validity. The number of deaths from rabies in the human family in our State is so inconsiderable in the last ten years that radical legislation of this kind is not received with the greatest favor.

Another bill which has been introduced is one of great value and merit, and should have the assistance and hearty cooperation of every veterinarian in our State. It is to make compulsory the reporting to the Livestock Sanitary Board every outbreak of contagious or infectious diseases among animals, with such data as will enable our State authorities to be fully conversant at all times with the condition of health of all our animals. It provides that these records shall only be made public when they can serve the interests of a community or the people of our State, and provides penalties for a failure to comply with its provisions.

Another bill has been introduced to provide for the safe destruction or disposal of the carcasses and of the parts and products of animals afflicted with certain dangerous or virulent diseases. This bill is destined to safeguard man or other animals against the possible dangers of consumption from these carcasses or products; to control the dangers incidental to their handling, slaughtering, or transportation. The best methods for robbing them of danger—incineration, burial, or the rendering-tank—are indicated by the cause for such condemnation or confiscation. Punishment is provided for failure to report, dispose of, or comply with the provisions of the Act.

This measure is destined to make clear and explicit the duty of owners and others in this direction, and does not leave open certain dangerous options that might suit the commerce, pleasure, or profit of an owner of such products and add an additional sanitary safeguard of such merit as should commend it to the thoughtful support of every veterinarian and good citizen of our Commonwealth.

In closing this report permit me to suggest for the consideration of our State Livestock Sanitary Board the preparation of, in the form of a small book, a digest of all the laws pertaining to the work of this Board and a ready-reference guide to the members of our profession who are ever solicitous to coöperate with the same in the excellent work already done and the wise, broad, conservatve plans inaugurated by our Board, and which promises for the grand old Keystone State a record in this direction unattained by any of our sister commonwealths.

W. Horace Hoskins, D.V.S.,
Chairman.

